# A New Ultrafine Luminescent La_2_O_3_:Eu^3+^ Aerogel

**DOI:** 10.3390/gels9080615

**Published:** 2023-07-29

**Authors:** Víctor M. García Ramírez, Antonieta García Murillo, Felipe de J. Carrillo Romo, Rosa I. Alvarez González, Eduardo Madrigal Bujaidar

**Affiliations:** 1 Instituto Politecnico Nacional CIITEC, Azcapotzalco, Mexico City 02250, Mexico; vgarciar1700@alumno.ipn.mx (V.M.G.R.); angarciam@ipn.mx (A.G.M.); 2 Instituto Politecnico Nacional ENCB, G.A.M, Mexico City 11350, Mexico; ralvarezg@ipn.mx (R.I.A.G.); emadrigalb@ipn.mx (E.M.B.)

**Keywords:** aerogels, rare earths, luminescence

## Abstract

This paper reports on the synthesis and characterization of La_2_O_3_:Eu^3+^ luminescent aerogels fabricated by the sol–gel method and the supercritical drying technique. The % mol concentration of the Eu^3+^ ion was varied to study the effects on the luminescent properties of the aerogels. XRD and TEM analysis showed that the La_2_O_3_:Eu^3+^ aerogels exhibited a semi-crystalline behavior regardless of whether the concentration of europium was increased. SEM micrographs revealed a porous structure in the aerogels, which were composed of quasi-spherical nanoparticles that were interconnected and formed coral-shaped agglomerates. Photoluminescence spectroscopy characterization showed that the aerogels had an infrared emission located at λ = 793 nm, and the maximum photoluminescence emission intensity was observed for the aerogel with 50% Eu^3+^. The results demonstrate that, without heat treatment, it is possible to manufacture luminescent aerogels of rare-earth oxides that can be used in opto-electronic devices.

## 1. Introduction

Due to the properties of aerogels, such as a large surface area and small particle size [1,2], in addition to the fact that they can be made from metal oxides (for example, rare-earth oxides) [3], they have aroused great interest for use in photoluminescent applications [4,5], mainly as nanophosphors. Phosphors (inorganic luminescent materials) have been extensively studied for their use in displays and are currently used in high-definition television displays (HDTV), plasma displays (PDP), cathode ray tubes (CRT), and field emission displays (FED) [6,7]. As regards the synthesis of phosphors, three groups of variables are studied and controlled for: (a) morphology and particle size, (b) stoichiometry and composition, and (c) surface chemistry [8,9]. Because phosphors can be used in high-resolution applications, nanometric particles with a spherical morphology and homogeneous composition are highly prized [10,11]. Shea et al. [12] observed that the luminescence intensity of Y_2_O_3_:Eu^3+^ phosphors increased if the reaction temperature was increased, which in turn led to an increase in the crystallite size of the material. Jung et al. [13] found that Y_2_O_3_:Eu^3+^ particles with a small surface area showed higher luminescence than those with a large surface area and that the luminescence intensity was directly proportional to the crystallite size. This trend has also been observed in other phosphors, such as Gd_2_O_3_:Eu^3+^ [11]. Wang et al. [9] demonstrated that a spherical morphology is required if one wishes to improve the emission intensity of phosphors prepared by the spray pyrolysis route. On the other hand, Zhang et al. [14] were able to synthesize Gd_2_O_3_ aerogels using the sol–gel method and the CO_2_ supercritical drying technique to obtain transparent aerogels. The XRD results showed that the aerogels were amorphous, and the nitrogen adsorption/desorption analysis showed a surface area of 223 m^2^/g, an average pore diameter of 42 nm, and a large pore volume of 1.83 mL/g. Similarly, Worsley et al. [15] fabricated chlorine-free rare earth oxide aerogels from the lanthanide series using a modified epoxide-assisted sol–gel method and a CO_2_ critical point dryer. All the aerogels were amorphous but became nanocrystalline after calcination at 923 K in air. The aerogels had high surface areas (up to 150 m^2^/g) and low densities (40–225 mg/cm^3^), and the Eu_2_O_3_, Tb_2_O_3_, Sm_2_O_3_, and Nd_2_O_3_ aerogels were photoluminescent. Cabrera et al. [16] synthesized TTA/Er_2_O_3_/Eu_2_O_3_ aerogels using the sol–gel method and supercritical drying with CO_2_. The SEM analysis showed that the TTA/Er_2_O_3_/Eu_2_O_3_ aerogels consisted of agglomerates of irregularly shaped particles ranging from 100 nm to 1 μm in size. These aerogels showed emissions when excited at λ = 613 nm.

A wide variety of methods are employed in the synthesis of phosphors, such as the hydrothermal method [17], spray pyrolysis [18], precipitation [19], and the sol–gel method, with a variation of the sol–gel method known as epoxide-assisted gelation [20], and, along with a low-temperature supercritical drying technique [3], aerogels can be made. One of the advantages of the sol–gel method is that it is possible to control the size and morphology of the particles, as well as their chemical composition [21], so that it is feasible to manufacture a luminescent rare earth oxide aerogel that is a nanophosphor.

Among luminescent materials, doped rare earth phosphors—for example—phosphors doped with Eu^3+^ ions, which emit a red color, are of technological importance because they are widely used in color displays and fluorescent lamps [6,7,8,9,10,11]. Another example, Eu^3+^-doped yttrium oxide (Y_2_O_3_:Eu^3+^), is considered one of the best red phosphors currently available [22]. One of the most common ions used in luminescent applications is the Eu ion, mainly because its spectroscopy is very well described. In its II and III oxidation states, this element produces blue (448 nm) light emissions in the case of Eu^2+^ and red (613 nm) in the case of Eu^3+^ [23]. These properties make europium compounds suitable for use in many devices and fields of application, for example, in optical fibers [24], photo-storage [25], lasers [26], biological markers [27], and inorganic light-emitting diodes (OLED) [28]. Both cations (Eu^2+^ and Eu^3+^) have a weak luminescence intensity, although it has been observed that this intensity increases when the particles are nano-sized [11]. One way to increase the light intensity of these ions is by inserting them into inorganic compounds (such as La_2_O_3_), which promotes a transfer of energy to the metal ions and a breaking up of their structural symmetry, leading to an increase in emission intensity [29]. Also, lanthanum oxide has numerous industrial and technological applications [30,31], including use in luminescent displays and light-emitting diodes (LED) [32], and La^3+^ ions can be easily replaced with luminescence-active Ln^3+^ ions over a wide range of concentrations [33], due to their ionic radius, electronegativity [34], and electron structure [35], much like the other lanthanide ions. La_2_O_3_ is recognized as an excellent host material for lanthanides in luminescence-related applications [36], and compared to other lanthanide host matrices, such as Gd_2_O_3_ and Lu_2_O_3_, it is cheaper and, therefore, lends itself to more industrial and technological applications [37,38]. 

In this study, a series of novel luminescent La_2_O_3_ aerogels doped with Eu^3+^ were synthesized by the sol–gel method via epoxide-assisted gelation and the low-temperature supercritical drying technique (with CO_2_). To obtain the highest luminescence intensity, the concentrations of the Eu^3+^ ion were varied at 2%, 5%, 8%, 10%, 20%, 30%, 40%, and 50% mol within the La_2_O_3_ host matrix. This could be performed because the spectroscopic properties of the Eu^3+^ ion are well known. The aerogels were characterized by XRD, TEM, SEM, FT-IR, and PL analyses to determine their spectroscopical, structural, and morphological properties. Photoluminescent emission (PL) characterization was performed at room temperature using an excitation of 394 nm.

## 2. Results and Discussion

### 2.1. Crystal Structure and Porous Texture

**Figure 1:**
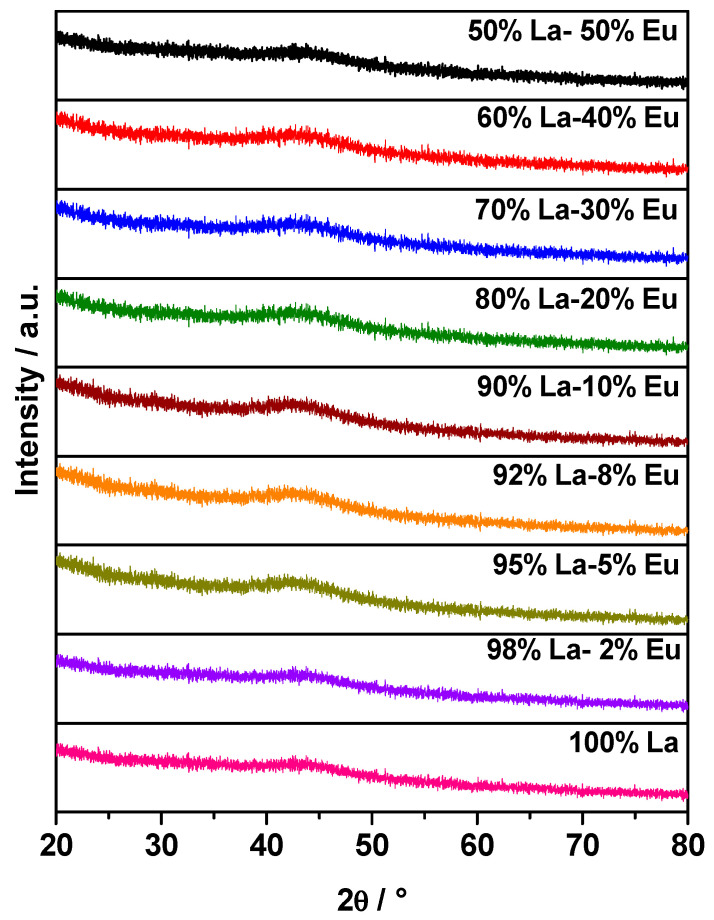
Diffraction peaks of the La_2_O_3_ aerogels at different concentrations of Eu^3+^.

Figure 1 shows the X-ray diffraction (XRD) patterns of the La_2_O_3_ aerogels. It can be seen that the aerogels are amorphous. However, a slight peak was observed around 43°, so a HRTEM analysis was performed. 

**Figure 2:**
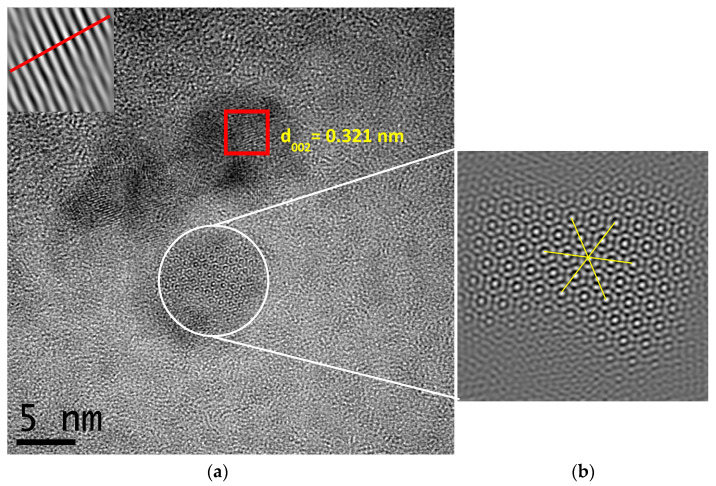
(**a**) HRTEM micrograph of La_2_O_3_ aerogel with 50% Eu^3+^; (**b**) image edited with DigitalMicrograph.

Figure 2a shows the HRTEM image for the 50% Eu^3+^ aerogel, whose lattice fringes show the crystallographic plane [002] with interplanar spacing of 0.321 nm, according to the ICDD 01-083-13-54 file of La_2_O_3_ with a hexagonal structure. It can be seen that the aerogels exhibit semi-crystalline behavior. A material can become crystalline if the temperature and pressure conditions are adequate, so the semi-crystalline nature of the aerogels is likely due to the pressure used in the supercritical drying. Figure 2b, an image edited with DigitalMicrograph software, shows an ordered region made up of multiple hexagons that repeat and form larger hexagons, something never seen before in an aerogel.

**Figure 3:**
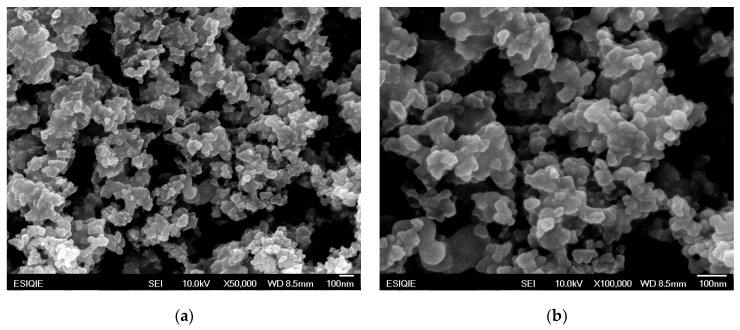
(**a**) SEM micrograph of the La_2_O_3_ aerogel with 50% Eu^3+^ at ×50,000 magnification; (**b**) SEM micrograph of the La_2_O_3_ aerogel with 50% Eu^3+^ at ×100,000 magnification.

Figure 3 shows SEM images of the 50% Eu^3+^ monolithic aerogel. The micrographs of the La_2_O_3_ aerogel with 50% Eu^3+^ reveal a non-ordered porous material composed of nanoparticles that form coral-shaped agglomerates; these nanoparticles are of varied sizes and are interconnected, forming a 3D network. 

**Figure 4:**
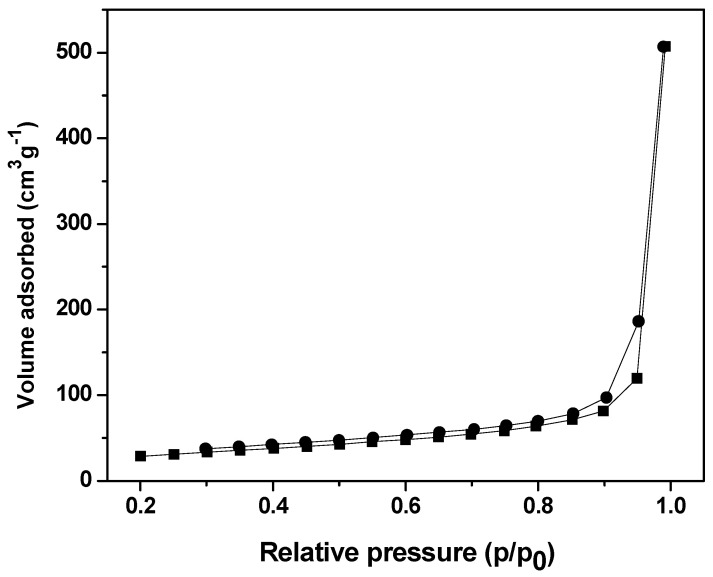
N_2_ adsorption—desorption isotherm.

**Figure 5:**
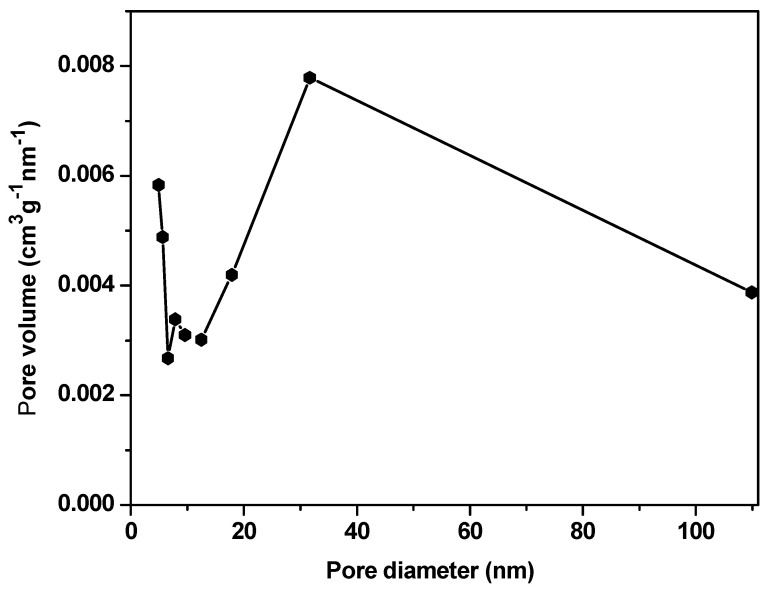
BJH pore size distribution curve of the sample.

The nitrogen adsorption/desorption technique was used to measure the pore volume, the average pore diameter, and the surface area of the La_2_O_3_:Eu^3+^ aerogel. According to Brunauer et al., it is a type IV isotherm with a H4 hysteresis loop [14,39]. The results are shown in Figure 4. Its surface area is 109.235 m^2^·g^−1^, as the average pore diameter and pore volume are 31 nm and 0.750 mL·g^−1^, respectively (Figure 5). 

**Figure 6:**
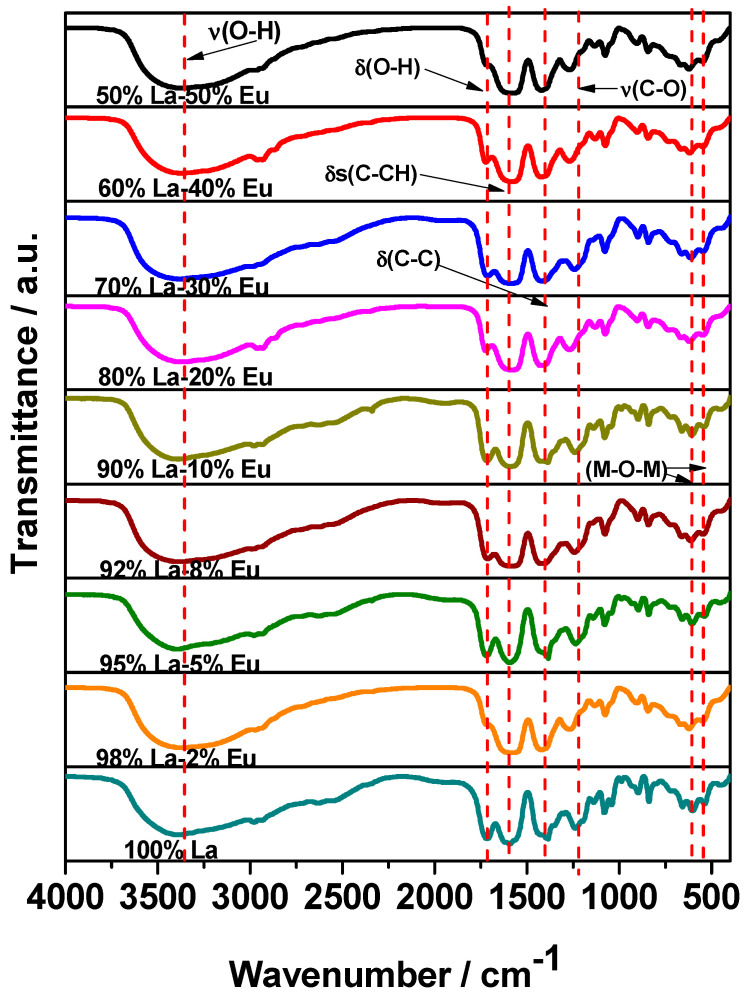
FT-IR spectra for the La_2_O_3_ aerogels at different concentrations of Eu^3+^.

Figure 6 shows the FT-IR spectra of the La_2_O_3_:Eu^3+^ aerogels at different concentrations of Eu. The spectra exhibit a broad band centered at 3400 cm^−1^, which is associated with the O–H stretching vibrations of water molecules. The absorption bands centered at 1635 cm^−1^, 1430 cm^−1^, and 1260 cm^−1^ can be attributed, respectively, to *δ*(O–H) deformation vibrations; *δ*s(C–H) oscillations, due to the deformation of the ethanol molecule; and *δ*(C-C), a bond associated with the propylene oxide used for the synthesis. Other absorption bands, centered at 1098 cm^−1^, 610 cm^−1^, and 560 cm^−1^, are associated with the stretching of υ(C–O) and with M-O m vibrations [3,40,41].

### 2.2. Photoluminescent Properties 

**Figure 7:**
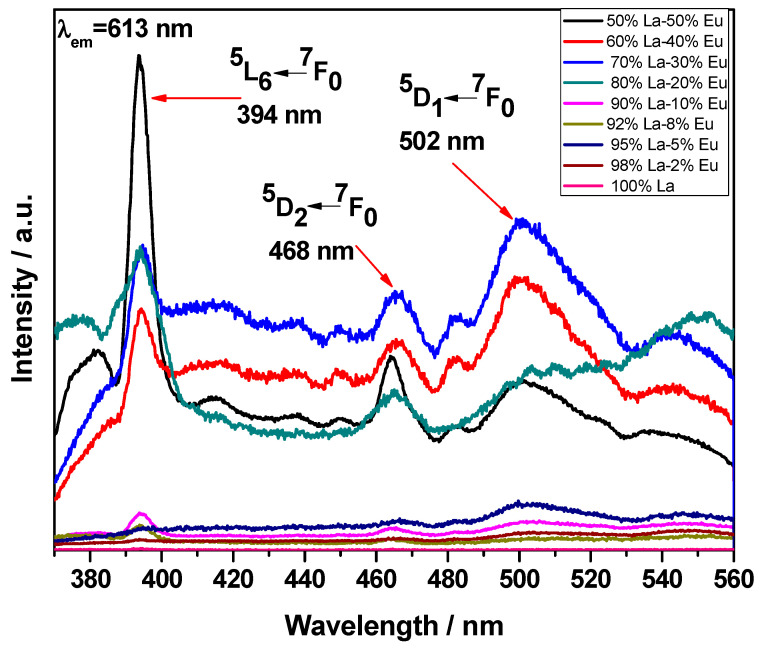
Excitation spectra of the La_2_O_3_ aerogels at different concentrations of Eu^3+^.

Figure 7 shows the excitation spectra of the La_2_O_3_ aerogels at different concentrations of Eu^3+^. The excitation spectra were measured in the range from 200 to 560 nm with λ_em_ = 613 nm. Maximum absorption can be seen at 394 nm, corresponding to the ^5^L_6_ ← ^7^F_0_ transition. Other absorption bands are located at 468 nm and 502 nm, corresponding to the ^5^D_2_ ← ^7^F_0_ and ^5^D_1_ ← ^7^F_0_ transitions, respectively. Note that the intensity of the charge transfer band (CTB) is lower than the maximum absorption peak located at 394 nm. This lower intensity of the CTB with respect to the transition ^5^L_6_ ← ^7^F_0_ was due to the lack of oxygen linked to La_2_O_3_ and to the weak interaction between the Eu^3+^ and O^2^- orbitals [42]. Even at high concentrations of Eu, no displacement of CTB was observed, which suggests that the crystalline environment around the Eu ions in the La_2_O_3_:Eu^3+^ aerogels was not substantially affected [42], since the CTB is normally displaced toward longer wavelengths due to changes in the crystalline field around the Eu dopant [43]. The electric dipole ^5^L_6_ ← ^7^F_0_ transition is the strongest transition in the excitation spectrum of the Eu^3+^ compounds, except when a ^7^F_6_ ← ^7^F_0_ transition in the near infrared region is observed [44]. This transition is commonly used to excite Eu^3+^ and induce photoluminescence if excitation through ligands is not possible due to a lack of efficient energy transfer. The excitation at the ^5^L_6_ level allows the 4f levels to be populated directly. It is worth noting that the transition ^5^D_0_ → ^7^F_4_ in yttria aerogels is particularly strong and not split [41]. The ^5^D_1_ ← ^7^F_0_ transition is a magnetic dipole transition, while the ^5^D_2_ ← ^7^F_0_ transition is a hypersensitive electric dipole transition (∆J = 2) [43].

**Figure 8:**
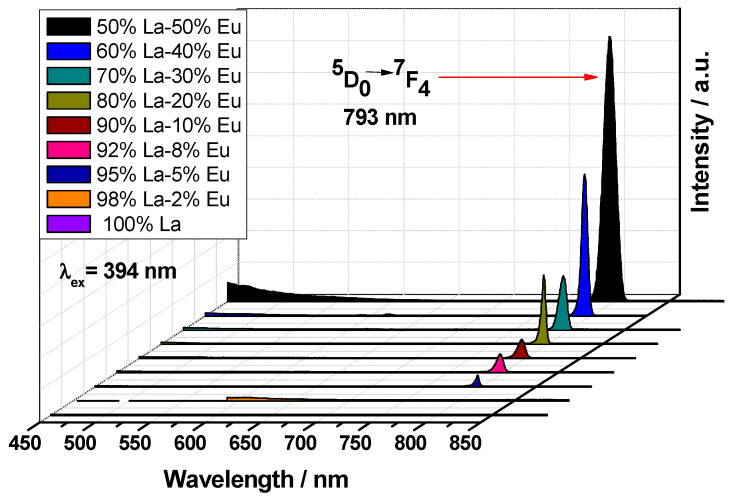
Emission spectra of the La_2_O_3_ aerogels at different concentrations of Eu^3+^.

The down-conversion (DC) luminescence spectra of La_2_O_3_ aerogels at different concentrations of Eu^3+^ are shown in Figure 8. The emission spectra were measured in the range from 450 to 900 nm, with *λ*ex = 394 nm. An infrared emission band can be observed at 793 nm, corresponding to the ^5^D_0_ ← ^7^F_4_ transition. This transition is sometimes considered hypersensitive because it does not obey the selection rules for quadrupole transitions (∆J ≠ 2); the intensity of this transition is determined by symmetry factors and by the chemical composition of the host matrix [44]. The luminescence spectra of compounds with D_4d_ symmetry are often dominated by the ^5^D_0_ → ^7^F_4_ transition since, with this symmetry, the ^5^D_0_ → ^7^F_2_ transition is forbidden, and the ^5^D_0_ → ^7^F_4_ transition is intense, because there is no center of symmetry [39,44]. Since the La_2_O_3_:Eu^3+^ aerogels have a strong emission only at 793 nm, we can assume that the Eu ions might be situated at a symmetrical site.

**Figure 9:**
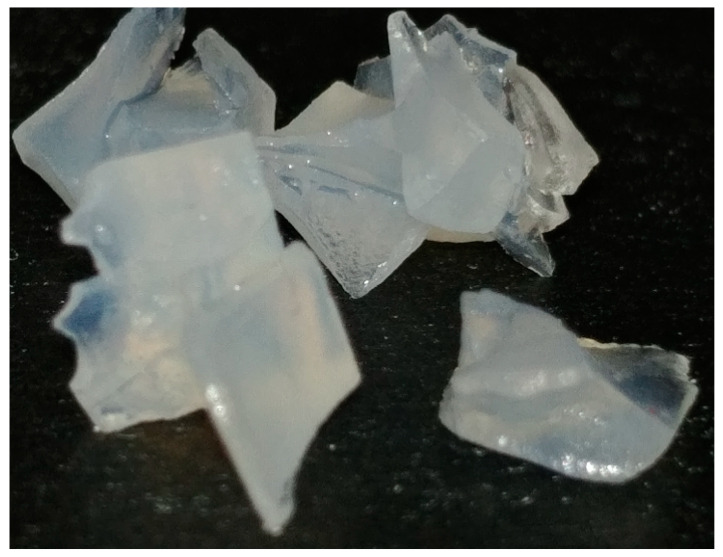
La_2_O_3_ aerogel with 50% Eu^3+^.

An increase in the luminescent center concentration should be accompanied by an increase in the intensity of emitted light due to higher absorption efficiency [33]. In Figure 8, it can be observed that the aerogel with 50% mol of Eu^3+^ has the highest luminescence intensity. This suggests that the Eu^3+^ ion does not act as a luminescence sensitizer in a La_2_O_3_ matrix; however, the La^3+^ ion does act as a luminescence sensitizer of the Eu^3+^ ion. An ordinary photograph of the La_2_O_3_:Eu^3+^ aerogel is show in Figure 9.

## 3. Conclusions

In this study, the monolithic aerogels synthesized by the sol–gel method via epoxide-assisted gelation exhibited a non-ordered 3D porous structure composed of interconnected nanoparticle agglomerates. Although no heat treatment was applied to the aerogels, they exhibited semi-crystalline behavior due to the high pressure used during supercritical drying. On the other hand, a change in the luminescence intensity was seen as the concentration of the dopant ion was increased, and the highest photoluminescence emission intensity was observed for the La_2_O_3_ aerogel with 50% mol Eu^3+^; this increase in luminescence intensity was probably due to a higher concentration of luminescent Eu^3+^ ions, which made them more likely to be directly excited at the wavelength used.

## 4. Materials and Method

The La_2_O_3_:Eu^3+^ aerogels were synthesized using the sol–gel method with epoxide-assisted gelation and the low-temperature supercritical drying technique. This approach offers many advantages in the preparation of metal oxide aerogels. First, this technique utilizes simple metal salts (e.g., metal nitrates or halides) as precursors in the sol–gel reaction, eliminating the need for organometallic precursors, such as metal alkoxides (more expensive, difficult to obtain, and very unstable). In addition, the process is flexible and allows for control over the microstructure of the gel network through modification of the synthetic parameters. Moreover, one of the advantages of epoxide-initiated gelation is that this approach provides a versatile and relatively straightforward route to the preparation of binary or ternary oxides. Like single-component systems, mixed metal oxide gels can be readily prepared through the addition of epoxides to solutions containing two or more metal salts. When synthesizing these mixed-metal oxide aerogels, hydrolysis and condensation reactions can yield a variety of different network architectures within the aerogel framework. For example, the condensed phase can be comprised of separate interpenetrating networks of the two metal oxides, –M1–O–M1– and –M2–O–M2–, or mixed phases of the two materials, –M1–O–M2–. Alternatively, one of the metal oxides can exist as discrete entities (i.e., nanoparticles) supported by the primary oxide structure. In general, the composition and bonding motif of the gel structure are primarily functions of the reaction stoichiometry of the inorganic precursors and the relative rates of hydrolysis of the metal ions [3,45]. In this work, the concentrations (molar percentages) of Eu^3+^ ions were varied to study the effects on the luminescent properties of a La_2_O_3_ aerogel. 

Lanthanum oxide (La_2_O_3_, 99.9%, Sigma-Aldrich) and europium oxide (Eu_2_O_3_, 99.9%, Sigma-Aldrich) were used as precursors in the synthesis. Ethanol (EtOH, CH_3_CH_2_OH, 99.9%, Fermont) was used as a solvent, propylene oxide (C_3_H_6_O, 99%, Sigma-Aldrich) as a gelation initiator, and granulated monohydrate citric acid (C_6_H_8_O_7_·H_2_O 99%, J.T. Baker) as a chelating agent.

The oxide precursors were changed into their respective chloride forms by reacting with hydrochloric acid, while being subjected to agitation until the solution became transparent. Once this occurred, 34.6 mmol of ethanol was added, and the mixture was stirred (300 rpm) for 5 min to homogenize it. After that, 7.5 mmol of propylene oxide was added to the solution and stirred (300 rpm) for another 5 min. Subsequently, 18.1 mmol of an alcoholic solution of citric acid (0.1 molar) was added, stirred for 2 min, and poured into cylindrical plastic containers until the mixture gelled. Once the wet gel was obtained, enough ethanol (EtOH, CH_3_CH_2_OH, 99.9%, Fermont) to cover the wet gel was poured into the container to begin the gel aging process, which lasted 24 h. After this, the wet gels were placed for 24 h (what is the time required to produce an exchange between the ethanol into the wet gels and the CO_2_) inside an E3100 critical point dryer filled with liquid CO_2_ and the CO_2_ brought to supercritical condition (32 °C and 74 bars). Later, the dryer was heated to 40 °C and pressurized to 83 bars, and it was kept at those settings for 1 h. After that, the temperature was raised to 50 °C, and the dryer was pressurized to 97 bars for 30 min. Finally, it was depressurized over the course of 1 h. 

The fluorescence emission was analyzed using Hitachi model F-7000 equipment with a 150 W xenon lamp and a R928F photomultiplier tube. The output slit was set at 5, as was the input slit, and the wavelength scanning speed was 1200 nm·min^−1^. The system was controlled by PC, using FL-Solutions software. The equipment used for the FT-IR analysis was a Perkin-Elmer model Spectrum 65 in the range from 4000 to 400 cm^−1^ with a speed of 5 scans per minute. The system was controlled by PC using PerkinElmer Spectrum software. For this test, the KBr filling method was also used. The crystalline structure of the powders was identified with a Bruker model D8 Advance Eco powder diffractometer with a Cu tube (1.5418 Å), and a model SS160 high-speed detector. The 2θ analysis interval was established from 20° to 80° with 0.01° increments and a sweep time of 0.2 s. The HRTEM images of the synthesized aerogels were captured with a Hitachi model S-3500N at a voltage of 5.0 kV and Jeol model JSM-7800F equipment operating with a vacuum at a voltage of 5.0 kV.

## Data Availability

The data that support the findings of this study are available on request from the corresponding author. The data are not publicly available due to privacy or ethical restrictions.
